# Electronic Cigarettes and Head and Neck Cancer Risk—Current State of Art

**DOI:** 10.3390/cancers12113274

**Published:** 2020-11-05

**Authors:** Marta Szukalska, Krzysztof Szyfter, Ewa Florek, Juan P. Rodrigo, Alessandra Rinaldo, Antti A. Mäkitie, Primož Strojan, Robert P. Takes, Carlos Suárez, Nabil F. Saba, Boudewijn J.M. Braakhuis, Alfio Ferlito

**Affiliations:** 1 Laboratory of Environmental Research, Department of Toxicology, Poznan University of Medical Sciences, 60-631 Poznan, Poland; 2 Institute of Human Genetics, Polish Academy of Sciences, 60-479 Poznan, Poland; szyfkris@man.poznan.pl; 3 Department of Otolaryngology, Hospital Universitario Central de Asturias-University of Oviedo, 33011 Oviedo, Spain; jprodrigo@uniovi.es; 4 Instituto de Investigación Sanitaria del Principado de Asturias, Instituto Universitario de Oncología del Principado de Asturias, University of Oviedo, CIBERONC, 33011 Oviedo, Spain; csuareznieto@gmail.com; 5 University of Udine School of Medicine, 33100 Udine, Italy; 6 Department of Otorhinolaryngology, Head and Neck Surgery, University of Helsinki and Helsinki University Hospital, 00029 HUS Helsinki, Finland; antti.makitie@hus.fi; 7 Research Program in Systems Oncology, Faculty of Medicine, University of Helsinki, 00014 Helsinki, Finland; 8 Division of Ear, Nose and Throat Diseases, Department of Clinical Sciences, Intervention and Technology, Karolinska Institute and Karolinska Hospital, 141 86 Stockholm, Sweden; 9 Department of Radiation Oncology, Institute of Oncology, 1000 Ljubljana, Slovenia; pstrojan@onko-i.si; 10 Department of Otolaryngology-Head and Neck Surgery, Radboud University Medical Center, 6525 GA Nijmegen, The Netherlands; robert.takes@radboudumc.nl; 11 Department of Hematology and Medical Oncology, Emory University, Atlanta, GA 30322, USA; nfsaba@emory.edu; 12 Member of the International Head and Neck Scientific Group, 35100 Padua, Italy; braakoncolres@gmail.com; 13 Coordinator of the International Head and Neck Scientific Group, 35100 Padua, Italy; profalfioferlito@gmail.com

**Keywords:** electronic cigarettes, toxicity, carcinogenic compounds, head and neck cancer, head and neck squamous cell carcinoma

## Abstract

**Simple Summary:**

The risk of developing cancer is always higher for tobacco smokers than for non-smokers. Electronic cigarettes (e-cigarettes) have become increasingly popular in the last decade and are considered less harmful than traditional tobacco products, due to the lower content of toxic and carcinogenic compounds. However, this is still a controversial issue. This paper contains a review of previous reports on the composition of e-cigarettes and their impact on the pathogenesis and risk of head and neck cancer (HNC). The authors reviewed articles on both toxic and carcinogenic compounds contained in e-cigarettes and their molecular and health effects on the upper respiratory tract in comparison to traditional tobacco cigarettes. In conclusion, the studies discussed in the review strongly suggest that more long-term studies are needed to better address the safety of e-cigarettes.

**Abstract:**

E-cigarettes have become increasingly popular in the last decade and are considered less harmful than traditional tobacco products due to the lower content of toxic and carcinogenic compounds. However, this is still a controversial issue. This paper contains a review of previous reports on the composition of e-cigarettes and their impact on the pathogenesis and risk of head and neck cancer (HNC). The objective of the review was to compare the molecular and health effects of e-cigarette use in relation to the effects of traditional cigarette smoking in the upper respiratory tract, and to assess the safety and effect of e-cigarettes on HNC risk. A review for English language articles published until 31 August 2020 was made, using a PubMed (including MEDLINE), CINAHL Plus, Embase, Cochrane Library and Web of Science data. The authors reviewed articles on both toxic and carcinogenic compounds contained in e-cigarettes and their molecular and health effects on the upper respiratory tract in comparison to tobacco cigarettes. The risk of developing head and neck squamous cell carcinoma (HNSCC) remains lower in users of e-cigarettes compared with tobacco smokers. However, more long-term studies are needed to better address the safety of e-cigarettes.

## 1. Introduction

In the last decade, the popularity of electronic cigarettes (e-cigarettes) has increased in comparison to the tobacco market [1]. They first appeared in Europe in 2005 and were mainly sold via the Internet. Since then, the e-cigarette market has been developing quite dynamically. It was not until 2014 that the European Parliament and United State Food and Drug Administration (FDA) developed regulations requiring standardization and quality control for e-liquids and vaporizers [2,3]. E-cigarettes are now available in tobacco shops and special stands. Their safety and efficacy in smoking cessation still remains controversial. In the general population, these products were considered attractive as an alternative to traditional cigarettes, due to their potentially less harmful effects and as a potential means to stop smoking [1,4,5,6]. However, a recent cross-sectional epidemiologic study covering 28 European Union (EU) countries demonstrated that dual users (cigarette smokers who also use e-cigarettes) smoke daily significantly more traditional cigarettes than smokers who are not e-cigarettes users. These results do not confirm that e-cigarettes have a positive impact on public health [7].

E-cigarettes are used mainly by tobacco smokers and former smokers [8,9]. It is estimated that 13% to 50% of tobacco smokers also use e-cigarettes. A survey including 27,801 Europeans reported that 67% use e-cigarettes to quit, 44% to smoke in places where smokers are banned, and 24% for the attractiveness and trendiness of their use [6]. Between 2016 and 2018, a significantly increasing prevalence of the e-cigarettes use in USA was found among middle-aged adults, women, and former smokers, but decreased among current smokers [10]. These products have also become fashionable among non-smokers and particularly worrying is that this applies mainly to young people. They are very popular due to the content of flavorings and the use of new technologies [11,12]. The study conducted by Cullen et al. among US adolescence (*n* = 19,018 participants), showed that current e-cigarette use was 10.5% (95% confidence interval (CI), 9.4–11.8%) among middle school students and 27.5% (95% CI, 25.3–29.7%) among high school students [13].

Until 2014, e-cigarettes in the European Union were not subjected to legal regulations, which caused significant inconsistencies in their composition (concentration of nicotine, presence of heavy metals and other substances specific for traditional tobacco). In addition, e-cigarettes had various technical disadvantages, such as cartridge leakage, which creates the risk of accidental overdose of nicotine and fluid intake when replacing cartridges [14]. Serious side effects reported by the FDA since 2008 regarding e-cigarettes have included pneumonia, confusion, convulsions, hypotension, second degree facial burns, chest pain, increased heart rate and congestive heart failure [14,15]. Other side effects included: headache, dizziness, migraine, dementia, drowsiness, sore throat, shortness of breath, abdominal pain, pleurisy and vision problems. The above summarized problems mainly concerned sudden poisoning or short-term exposure [14,15]. Thus far, no long-term exposure effects have been described.

In the European Union, around 700,000 people die prematurely each year from the consequences of tobacco smoking [16]. Therefore, an important task of health policy is to investigate how to prevent smoking and to encourage smokers to quit. Some data indicate that e-cigarettes can be used as a nicotine replacement therapy for smokers [17,18] but the World Health Organization (WHO) underlines that, while e-cigarettes might be less harmful than tobacco cigarettes, electronic devices still pose important health risks. As of 20 May 2016, EU countries must comply with the EU Tobacco Products Directive. The Directive includes regulations for electronic nicotine delivery systems and states that their packaging should provide information on health warnings, addictiveness and toxicity, and a list of all the substances the product contains, including the exact nicotine level (not exceeding 20 mg/mL) [2]. In addition, the Directive prohibits the advertising, promotion and sale of e-cigarettes to minors. Consequently, e-cigarettes have disappeared from the Internet and public places. E-cigarettes and cartridges are sold only in specially designed physical outlets. At present, they can only be used in designated places; a financial fine may be imposed on offenders. These regulations were necessary to increase the safety and quality of electronic nicotine consumption systems. However, they do not resolve all issues related to them. Many people are interested in whether e-cigarettes are safe, especially in a long-term perspective, and whether they can cause cancer. Although research so far has not provided a clear answer to these questions, experts believe that the negative long-term effects of smoking e-cigarettes only become apparent after many years [19,20]. The issue of e-cigarette vaping-associated pulmonary injury (EVALI) is already known about; at the beginning of 2020, The Center for Disease Control and Prevention (CDC) announced a nationwide EVALI outbreak [21]. Vitamin E acetate in tetrahydrocannabinol (THC) containing e-cigarettes is believed to play a significant role in EVALI pathogenesis, however, the evidence is insufficient to rule out the contribution of other chemical compounds [21,22,23]. The current public health crisis related to Covid-19 may exacerbate health effects of e-cigarettes vaping [22,24]. Moreover, the information relating the health consequences of secondhand exposure to the aerosols (SHA) from e-cigarettes is still empirically unknown. There is a significant need for additional research to better understand how these products affect health.

Head and neck cancers (HNCs) are a heterogeneous group of tumors, responsible for more than 650,000 cases and 330,000 deaths per year worldwide, and one of the most important etiological factors is tobacco smoking [25]. The first contact organ for vaporized e-liquids is mucosal tissue of the upper respiratory tract and the upper part of the digestive tract. E-liquids contain chemical compounds, cytotoxic to the upper airway tissue, and can cause significant DNA damage. Therefore, the mutagenicity of e-liquids cannot be completely excluded as a risk factor for HNC [26]. This article is a review and summary of existing reports on the impact of e-cigarettes on the incidence of HNC. 

A review for 163 English language articles published until 31 August 2020 was made, using a PubMed (including MEDLINE), CINAHL Plus, Embase, Cochrane Library and Web of Science data. Seventeen of these articles were directly related to studies on e-cigarettes and head and neck cancers. Among them, 11 were studies on e-cigarette liquid exposure based on various cell models (in vitro studies), 1 animal model study and 5 studies concerning the impact of aerosol from e-cigarettes on their users (in vivo studies). The literature search was performed using MESH terms and other relevant keywords. The key search terms were “electronic cigarettes” (or “e-cigarettes”) and “head and neck cancer”, “electronic cigarettes” and “head and neck squamous cell carcinoma”, “electronic cigarettes” and “head and upper respiratory tract”, “electronic cigarettes” and “tobacco smoke” and “head and neck cancer”, “electronic cigarettes” and “toxic and carcinogenic compounds”. Expert statements, recommendations, technical reports, other non-original papers, conference papers and preprints were excluded from our review. All of the presented original papers were peer-reviewed. Moreover, the authors attempted to assess the risk and potential mechanisms related to HNC resulting from the use of electronic cigarettes and smoking of traditional cigarettes.

## 2. Toxic and Carcinogenic Compounds in E-Cigarettes vs. Traditional Cigarettes

E-cigarettes consist of a drive unit, an electric atomizer and replaceable cartridges containing a liquid, which is sprayed and converted into an inhalable aerosol that is sucked in by the user by means of a mouthpiece. The main components of the liquids are propylene glycol and/or glycerin, aromas (e.g., with fruit, chocolate, rum, coca cola or tobacco flavors). Nicotine is the active substance in the e-cigarette liquid fillers, but they are also available in a nicotine-free form [4,5].

The degree of exposure to nicotine in aerosols of e-cigarettes varies considerably. Studies have shown a large discrepancy in the concentrations of this alkaloid among e-cigarette users. There are publications showing that the levels of cotinine (the main nicotine metabolite) in the saliva of e-cigarette users are similar to those of active smokers of conventional cigarettes [27,28].

Furthermore, until the effective date of the EU Directive (2016), reports indicated that manufacturers’ declarations on the composition of e-liquids varied from real ones, and consumers usually did not have authentic information on the quality of e-cigarette they were using [2]. Prior to the introduction of legal regulations, adulterations concerning nicotine concentrations were frequent. In one study, nicotine was detected in e-cigarette liquids in the range of 14.8–87.2 mg/mL and differed from the amount declared on the label by as much as 50% [29]. In an extreme case, nicotine was detected in non-nicotine fluid in the amount of 21.8 mg, which corresponds to fluids with high content of this compound [30]. 

In the absence of tobacco smoke, the effect of the long-term use of nicotine on cancer risk is not clearly understood. It is an important aspect in the evaluation of the possible long-term effects from sources of nicotine, such as e-cigarettes, which have a potential for life-long use. Generally, there exists inadequate evidence to conclude that nicotine per se may impact carcinogenesis in humans [31]. However, nicotine through nicotine acetylcholine receptors (nAChRs) activates signaling pathways that result, among others, in increased cell proliferation and cell survival [32,33]. It has been shown that nicotine induces chromosomal aberration and sister chromatid exchange, DNA single-strand breaks, formation of micronuclei in vitro and changes that mimic the effects of angiogenic growth factors, and may inhibit antitumor immune response [32]. The carcinogenic tobacco-specific N-nitrosamines (TSNA), which are known risk factors for HNCs, may be formed from nicotine in the body [32]. Thus, nicotine may enhance tumor progression in various ways. 

Compared to e-cigarettes, significant amounts of chemical compounds have so far been identified in tobacco smoke. Tobacco smoke is a mixture that contains over 5300 compounds [34]. The International Agency for Research on Cancer (IARC) indicated that 70 of them have carcinogenic activity [35]. Moreover, in accordance with IARC classification, 16 of these have been included into Group 1 of substances with proven carcinogenicity to humans [35]. Tobacco products for oral use definitively contain a number of carcinogenic substances, such as heavy metals, 4-(metylnitrosamino)-1-(3-pyridyl)-1-butanon) (NNK) and polycyclic hydrocarbons (PAHs), which undoubtedly contribute to tobacco related carcinogenesis in HNSCC [35]. The risk for HNSCC in smokers is approximately ten times higher than that of never smokers [36].

E-cigarettes were intended to be a healthier alternative to traditional tobacco products, but some studies have shown that apart from nicotine, in some e-liquids, other toxic and carcinogenic substances have also been found. In a Korean study evaluating 225 fluids purchased within one year, formaldehyde and acetaldehyde were present in all fluids [37]. Formaldehyde is a human carcinogen (Group 1) and poses nasopharyngeal cancer risk [38,39]. Based on epidemiological evidence, acetaldehyde associated with the consumption of alcoholic beverages is considered as a “Group 1 carcinogen” for the esophagus and/or head and neck [40]. Previous analysis of the e-liquids, aerosols and environmental emissions have showed that some contained trace levels of substances, namely, TSNAs, carbonyl compounds and PAHs [41,42]. The TSNAs and PAHs have been the most heavily studied tobacco smoke ingredients with regard to carcinogenicity. Of the seven TSNAs identified in tobacco smoke, NNK *N′*-nitrosonornicotine (NNN), and 4-(methylnitrosamino)-1-(3-pyridyl)-1-butanol) (NNAL) are potent carcinogens. The NNN reproducibly induces head and neck tumors in in vivo studies. Gavage or subcutaneous administration of NNN to laboratory animals produces predominantly nasal tumors. However, NNN administered in drinking water or diet causes oral, nasal and esophageal tumors [36]. The best studied carcinogenic constituents in PAH group include 1-hydroxypyrene (1-HOP) and benzo(a)pyrene. When given orally, PAH exposure results in tongue and esophageal cancers, and in tumors of the upper respiratory tract (larynx, trachea and nose), while inhalation exposure results in pharynx and esophagus tumors [36,43].

The presence of contaminants commonly present in tobacco such as anabasine, myosmine and β-nicotyrine with potentially harmful effects on humans, as well as heavy metals such as nickel, chromium and arsenic has also been detected in cartridges or aerosols of e-cigarettes, although most were found at much lower levels than in conventional cigarette smoke [29,30,41,44]. There is evidence that chronic exposures to heavy metals via tobacco smoking increase the risk of HNC, while less is known about the effects of e-cigarettes [35,45]. With regard to the presence of metals in e-cigarette liquids and aerosols, their concentrations may pose a significant cancer health risk [39]. Because the device design and heating elements are probably the main sources of metals in e-aerosols, the tightening of safety regulations and the improvement of production quality are necessary in order to reduce metal exposure in e-cigarette users [46].

In some reports, regarding electronic devices that simulate smoking, other volatile organic compounds such as benzene, xylene, toluene, and styrene were also detected at trace levels [42].

Moreover, the presence of other toxic substances (such as acrolein) have also been detected in e-cigarettes aerosols (0.07–4.19 µg per 15 puffs vs. 2.4–62 µg in mainstream smoke) [47]. Breathing even low levels of acrolein can irritate the nose, nasal cavity, pharynx and larynx [38].

The basic question is whether e-cigarettes are less harmful to health than traditional cigarettes? A number of ingredients and additives (such as vitamin E acetate) that appear in e-cigarettes—and their impact on health—have not been sufficiently studied (or not studied at all), which does not allow for a univocal statement concerning the safety of e-cigarettes. The International Union Against Tuberculosis and Lung Disease has issued a statement in which it points out that these products cannot be considered safe for second-hand exposure, as the e-cigarettes emits the finest particles of liquid nicotine and carcinogens into the air and consequently leads to inhaling them [48].

Furthermore, there are difficulties in establishing the reliable toxicity profile of e-cigarettes, since it may vary due to differences in design, depending on the manufacturer, the source and type of the ingredients used in their manufacture, and the means of production and quality control. However, the concentration of most substances measured in the aerosols or e-liquids of those products seem to be definitively lower than in the smoke from traditional cigarettes (Table 1).

There have been reports concerning interactions between environmental factors and virus-mediated epithelial carcinogenesis [49]. Some tobacco smoke compounds have been evaluated for carcinogenesis through molecular mechanisms involved in cooperation with high-risk human papillomavirus (HR-HPV), etiologically associated with HNC. Both tobacco smoke and HPV are involved in epithelial cancer development and progression, therefore a complex system of interactions can exist between them [49]. Moreover, it cannot be ruled out that similar mechanisms may also be observed in the case of e-cigarette users, but so far it has been not verified.

## 3. Interaction with Genetic Material

The first attempts to estimate the risk of developing cancer as a consequence of using e-cigarettes were associated with the determination and comparison of genotoxicants present in traditional tobacco smoke and vapors emitted by e-cigarettes. The factors taken into account as determinants of differences in the chemical compositions of tobacco smoke and e-cigarette vapor were as follows: combustion temperature equal to approximately 1000 °C in tobacco smoke vs. 250–300 °C vaporing temperature, a lack of side stream in e-cigarette, and controlled amount of nicotine in e-cigarette. The laboratories involved in the comparative studies explored the newest available chromatographic procedures for mixture separation (liquid or gas chromatography) and specialized techniques (e.g., mass spectrometry) for compound identification [41,42]. A general conclusion was a detection of some harmful substances in e-cigarette vapor identical with those present in tobacco smoke. The main point is that a majority of them were detected in trace concentrations or were below the detection limit [54]. The review paper by Löhler and Wollenberg [55] concludes that the concentration of harmful substances in e-cigarette vapor is 9–450 times lower than that in cigarette smoke.

The studies on the total comparative profiles of the chemical composition of tobacco smoke and e-cigarette vapor did not exclude further detailed evaluation of participation of individual harmful compounds. Determination of four N-nitrosoamines having strong carcinogenic potential has shown that their concentration exceeds roughly 10 times the results mentioned by Korean producers [56]. Pankow et al. [57] described the formation of a benzene ring by dehydration of propylene glycol and glycerol at boiling temperature. Although benzene is known as a cancer-risk toxicant, its concentration formed in e-cigarettes is not followed by a negligible risk. A similar research pathway was used to determine concentrations of acetaldehyde, acrolein and formaldehyde in e-cigarette aerosol. Ogunwale et al. [58] noticed differences between different sorts of e-cigarettes, detecting higher levels in e-cigarettes using stronger batteries. Nevertheless, the concentration of acetaldehyde and acrolein was 100–1000 times lower than in tobacco cigarettes. The concentration of formaldehyde was 2–200 times lower than in tobacco cigarettes [58]. In association with the latter finding, attention was laid on flavoring compounds as the main contributors of aldehyde production in e-cigarettes aerosol [59].

The occurrence of nicotine and metabolites aerosol was not surprising in e-cigarettes, where producers declared its usage. The only exception concerns metals, with a special attention on nickel, with a proven carcinogenicity towards lung, nasal cavity and sinus. Aherrera et al. [60] have found considerable levels of nickel in urine, saliva and exhaled breath. Unfortunately, the control group of tobacco smokers was not included in the study.

Altogether, the concentration of chemicals inducing adverse health effects was found to be smaller in e-cigarettes than in tobacco cigarettes. An open question is whether such small amounts of (geno) toxic substances are still capable of inducing adverse health effects. Having established a reduced exposure to biologically active toxicants in e-cigarette users, it became necessary to estimate the biological effects of vaping.

As tobacco smoking induces oxidative stress, a few laboratories attempted to estimate its relation to e-cigarettes. According to in vitro study by Taylor et al. [61] on human bronchial epithelial cells, e-cigarette aerosol induces oxidative stress that is lower as compared with tobacco smoke. Ganapathy et al. [62] established significant oxidative DNA damage, as a result of antioxidant system suppression (decreased total antioxidant capacity (TAC) and 8-oxoguanine DNA glycosylase expression of (OGG1)) in human oral and lung cells, induced by e-cigarette aerosol extracts. Generation of reactive oxygen species by e-cigarette aerosol is followed by DNA damage and cell death in vascular endothelial cells [63]. In the study on two human monocytic cell lines by Muthumalage et al. [64], oxidative stress was attributed to flavoring chemicals added to e-cigarettes. The generation of single strand DNA breaks could result from reactive oxygen species as well as such carcinogens as carbonyl compounds. An exposure of a keratinocyte cell line and two head and neck cell lines (tumor and non-tumor) to e-cigarette vapor has shown an induction of DNA strand breaks in all three cell lines. The effect was not dependent on nicotine occurrence in the vapor [65]. DNA damage cannot be easily removed as exposure to e-cigarette aerosol reduces DNA repair activity in human and mouse cells derived from heart, lung and bladder [66].

When discussing the cellular effects of e-cigarette vapor exposure, the study of Azzopardi et al. [67] should be mentioned. Using human lung epithelial cells, the authors concluded that cytotoxicity induced by e-cigarette aerosol is much smaller than that induced by tobacco smoke. More detailed analysis was provided by Behar et al. [54] who tested three types of human cells (i.e., pulmonary fibroblasts, lung epithelial cells and embryonic stem cells) for cytotoxicity of refilling liquids and aerosols released from e-cigarettes. Cytotoxicity was demonstrated in all three tested cell lines with particularly high effect in stem cells. Unfortunately, a comparison with tobacco cigarettes was not included in the study.

The presence of carcinogens in the body fluids of e-cigarette users [68,69] implicates by itself that cells are at risk for carcinogenic transformation. Particularly, the study of Fuller et al. [69] is bringing a strong message, because of an identification of two carcinogens in the urine of e-cigarette users, although in a substantially lower concentration than in tobacco cigarette smokers. O-toluidine and 2-naphthylamine are suspected to act as bladder carcinogens in humans. Casual carcinogenic activity of 2-naphthylamine could be broader and not limited to the urinary tract. One of the recent studies proved that two months of e-cigarettes exposure as well as traditional cigarettes decreases bone marrow hematopoietic progenitor cells populations, which play a pivotal role in maintaining the body’s steady-state blood and immune cell populations [70].

Nevertheless, the literature data seem to exclude further mutagenic effect of e-cigarette components. Thorne et al. [71] assessed the mutagenic activity of e-cigarette aerosol using the commonly accepted Ames test, performed on two *Salmonella typhimurium* strains. As a reference, the mainstream smoke of Kentucky reference (3R4F) cigarette was applied. Freshly generated e-cigarette aerosol was found negative in both strains. In another experimental attempt, detecting mutation in the cII transgene that was taken as a reporter gene, only a limited mutagenicity was found in mouse and human cells exposed in vitro [72].

## 4. Biological Effects and Risk on Cancer

Inhaled e-cigarette aerosol will transport many substances through the mouth and throat into the lungs. There are known reports of oral and throat irritation, dry cough, increased respiratory impedance and resistance to respiratory flow similar to those after 21 cigarettes. Nicotine from aerosol or e-cigarette liquid may remain on the surface for several weeks or even months and react with the environment to form TSNA compounds with nitric acid, resulting in inhalation, ingestion or dermal exposure to carcinogens [26,32,73].

### 4.1. The Impact of E-Cigarette Liquid Exposure Based on Cell Models (In Vitro Study)

Some evidence exists from basic research, which used cells derived from the oral cavity [62,74,75,76,77,78,79], a panel of normal epithelial and head and neck squamous cell carcinoma cell lines [64], cultures of oropharynx [26] and of the middle ear [80] to investigate the effect of e-cigarettes on these cells (Table 2). Several of these studies demonstrated a cytotoxic effect of e-cigarettes [26,65,75,76,77,78,80] and varied extend of DNA damage [26,62,65], as well as intensified oxidative stress [62,64,75,77]. In cells exposed to e-cigarette vapor, a dose-related increase of DNA damages was observed, although lower than those induced by tobacco smoke extracts. Moreover, it has been shown that exposure to e-cigarette aerosols suppressed the cellular antioxidant defenses, through elevated reactive oxygen species (ROS) level and increased presence of 8-oxo-2′-deoxyguanosine (8-oxo-dG), one of the major products of DNA oxidation [62]. Another in vitro study demonstrated a high level of DNA strand breaks, elevated apoptosis, necrosis and cell death following exposure to e-cigarette aerosols, regardless of nicotine content [65]. In oropharyngeal mucosal cells exposed to e-liquids, a significant reduction in cell viability as well as increased DNA fragmentation were confirmed. The fruit-flavored liquids showed a higher toxicity than tobacco-flavored liquid [20].

### 4.2. The Impact of E-Cigarette Liquid Exposure in Animal Models (In Vivo Studies)

Only one of the in vivo studies regarding e-cigarettes was dedicated to checking their effect on laryngeal mucosa (Table 3). Salturk et al. [81] conducted and experimental study to explore the impact of e-cigarettes aerosol on the laryngeal mucosa of rats. Following four weeks exposure to e-cigarette aerosols, they observed, not statistically significant, hyperplasia and metaplasia of the laryngeal mucosa [81].

### 4.3. The Impact of Aerosol from E-Cigarette on Their Users (In Vivo Studies)

Only a few studies have examined the impact of e-cigarette liquid exposure on e-cigarette users (Table 4). In the cohort study of Franco et. al. [82], which involved 65 participants divided into three groups (non-smokers, tobacco smokers and e-cigarette users), the cytologic examination of the oral mucosa scrapings was carried out, however, no statistically significant differences were observed in micronuclei distribution between groups. Bustamante et al. [83] observed endogenous formation of the tobacco-specific oral and esophageal carcinogen *N′*-nitrosonornicotine (NNN) in e-cigarette users. Another study [84] investigated the effect of e-cigarettes (with and without nicotine) on blood flow in the buccal mucosa in 10 subjects immediately after vaping. The authors concluded that e-cigarettes may have an effect on blood flow to the oral mucosa, although further studies are needed. Bardellini et al. [85] performed a prospective case-control study concerning the prevalence and characteristics of oral mucosal lesions in ex-smokers (*n* = 45), compared to e-cigarettes users (*n* = 45) [85]. No statistically significant differences were observed in terms of total prevalence of oral mucosal lesions between former smokers and e-cigarette consumers. However, nicotine stomatitis, a hairy tongue and angular cheilitis resulted to be significantly more common among e-cigarette consumers. A newer study, with 119 participants (never smokers, tobacco smokers, e-cigarette users) showed that exposure to aerosol of e-liquid modulates the oral microbiome and elevates the abundance of oral pathobionts, induces gum inflammatory responses and makes epithelial cells more common to infection [86]. In the oncology literature, there are reports indicating the possibility of a relationship between changes occurring within the human microbiome, inflammation and cancer development. Bacteria can contribute to cancer processes by producing toxins, carcinogenic metabolites and initiating chronic inflammation [87].

## 5. Conclusions

With the current state of knowledge, e-cigarettes should not be considered safe. In contrast to traditional cigarettes, there is no burning and hence fewer by-products. Although the main components of e-cigarettes (propylene glycol, glycerin, flavor and fragrance substances) are substances commonly used in the food industry, it does not necessarily mean that they are completely safe during repeated inhalation over long-term use [41,42].

Scientific evidence regarding the human health effects of e-cigarettes is limited. While e-cigarette aerosols may contain fewer toxicants than cigarette smoke, studies evaluating whether e-cigarettes are less harmful than cigarettes are inconclusive. One of the findings from the Office of the Surgeon General of the United States states that: even people diagnosed with cancer should benefit from quitting smoking. No e-cigarette has been approved by the FDA as a cessation aid. Despite e-cigarettes allure, an observational study found that e-cigarettes are not an effective tool for cancer patients to quit smoking. E-cigarette users were twice as likely to be chronic tobacco smokers [88,89]. Environmental concerns and issues regarding non-user exposure exist. The exact health impact of e-cigarettes, for users and the public, can only be determined with more available data.

There is some evidence suggesting a potentially carcinogenic role of e-cigarettes in the pathogenesis of HNCs presented in original papers (17). Several in vitro studies (11) indicated the cytotoxic effect of e-cigarettes [26,65,74,75,76,77,78,80] and variable extents of DNA damage [26,62,65], as well as intensified oxidative stress [62,64,75,77,79]. We observed a lack of properly designed animal experimental models. Only one in vivo study explored the impact of e-cigarette aerosols on the laryngeal mucosa of rats [81]. Only a few studies (5) investigated exposure to e-cigarettes in context of HNCs in population-based studies [82,83,84,85,86]. Most of the available articles report basic laboratory experiments or cohort studies with small sample sizes, and are limited in their design, methodology, and the used exposure time and lack of long-term follow-up.

Taking into account all the reviewed data, cancer risk associated with e-cigarette use has a rather postulative character and it has not yet been proven by broad epidemiological studies [90,91]. However, due to the typically long duration of carcinogenesis and the relatively short history of e-cigarette use, caution should be taken regarding the risk of cancer they may present. A clear need still remains for the development of new studies regarding e-cigarettes and their impact on HNC risk.

## Figures and Tables

**Table 1:** Comparison of mean concentrations of carcinogenic compounds contained in vapor of e-cigarettes vs. smoke of traditional cigarettes.

Chemical Class	Carcinogen	IARC Group *	Vapor Generated from E-Cigarette (per 15 puffs)	Smoke from Single Non-Filter Cigarette [50]	Relationship of Carcinogens to HNSCC **
PAHs and Heterocyclic Analogs	Benzo(a)anthracene Benzo(a)pyrene Dibenzo(a,h)anthracene	2A 1 2A	ND in 1–100 puffs (LOD = 0.37 ng) [41] ND in 1–100 puffs (LOD = 0.53 ng) [41] ND in 1–100 puffs (LOD = 0.62 ng) [41]	20–70 ng 20–40 ng 4 ng	Larynx Oral cavity [50,51]
Aromatic Amines	4-aminobiphenyl 2-naphthylamine	1 1	NQ in 1–100 puffs (LOQ = 0.05 ng) [41] NQ in 1–100 puffs (LOQ = 0.12 ng) [41]	2–5.6 ng 1–334 ng	
N-Nitrosamines	NNK NNN	1 1	ND–2.83 ng [47] ND–0.43 ng [47]	130 ng 200 ng	Nasal Oral cavity Oesophagus [50,51]
Volatile Hydrocarbons	Benzene 1,3–butadiene Isoprene Styrene	1 1 2B 2B	ND in 1–100 puffs (LOD = 0.17 µg) [41] ND in 1–100 puffs (LOD = 0.29 µg) [41] ND in 1–100 puffs (LOD = 0.41 µg) [41] 0.518 µg/1–100 puffs [41]	20–70 µg 20–75 µg 450–1000 µg 10 µg	
Aldehydes	Formaldehyde Acetaldehyde	1 2B	0.32–5.61 µg [47] 0.20–1.36 µg [47]	70–100 µg 18–1400 µg	Nasopharyngeal Nasal [50,51]
Phenols	Catechol Caffeic acid	2B 2B	ND in 1–100 puffs (LOD = 0.26 µg) [41] ND in 1–100 puffs (LOD = 2.39 µg) [41]	100–360 µg <3 µg	
Miscellaneous Organic Compounds	Acrylonitrile Vinyl chloride Ethylene oxide	2B 1 1	ND in 1–100 puffs (LOD = 0.32 µg) [41] ND in 1–100 puffs (LOD = 6.57 ng) [41] ND in 1–100 puffs (LOD = 0.36 µg) [41]	3–15 µg 11–15 ng 7 µg	
Metals and Inorganic Compounds	Arsenic Beryllium Cadmium Chromium Cobalt Lead Nickel Polon-210	1 1 1 1 2B 2B 1 1	NQ in 1–100 puffs (LOQ = 8.79 ng) [41] ND in 1–100 puffs (LOD = 9.36 ng) [41] ND–22 ng [47] 10.5 ng [52] ND in 1–100 puffs (LOD = 8.93 ng) [41] 3–57 ng [47] 11–29 ng [47] ND in 1–100 puffs (LOD = N/A) [41]	40–120 ng 0.5 ng 7–350 ng 4–70 ng 0.13–0.2 ng 34–85 ng ND–600 ng 0.03–1.0 pCi	Oral cavity [53]

* Classification of carcinogens according to IARC: Group 1—carcinogenic to humans; Group 2A—probably carcinogenic to humans; Group 2B—possibly carcinogenic to humans. ** Gaps indicate that there are no reports on this topic. Abbreviations: LOD—limit of detection; LOQ—limit of quantification; ND—not detected; NQ—not quantifiable.

**Table 2:** The impact of e-cigarette liquid exposure based on cell models (in vitro study).

Type of Cells	Characteristic of E-Liquid	Action	Reference
▪ Human middle ear epithelial cell (HMEEC) line	E-liquids (12 manufacturers)	HMEEC viability reduction even without the application of nicotine. E–liquids cytotoxicity affected by the flavoring agents.	Song et al., 2018 (South Korea) [80]
▪ Human epithelial normal bronchial cells (Nuli1); human oral squamous cell carcinoma (UM-SCC-1); human premalignant dysplastic oral mucosalkeratinocyte cells (POE9n).	E-cigarette vapor extracts (5)	Dose–related ↑ of DNA damage, regardless of nicotine content. Significantly ↑ ROS: ↓ TAC; ↓ expression of 8-oxoguanine DNA glycosylase (OGG1), an enzyme essential for the removal of oxidative DNA damage.	Ganapathy et al., 2017 (USA) [62]
▪ Human keratinocytes (HaCaTs)	E-cigarette aerosol extract (7 brands)	↓ keratinocyte antimicrobial activity. Cytotoxic to cells (necrotic cell death). Alerted macrophage and neutrophil antimicrobial function.	Hwang et al., 2016 (USA) [76]
▪ Normal human oral keratinocytes (NHOKs)	E-cigarette aerosol with different nicotine strength and flavors	Induced oxidative stress: significant ↓ of intracellular glutathione (GSH) levels. ↑ cytotoxicity in oral epithelial cells.	Ji at al., 2016 (USA) [77]
▪ Human gingival epithelial cells	E-cigarette aerosol	Altered cellular morphology. ↑ lactate dehydrogenase (LDH) activity. ↑ apoptotic cell numbers.	Rouabhia et al., 2016 (Canada) [78]
▪ Human periodontal ligament fibroblasts (HPdLF); human gingival epithelium progenitors pooled (HGEPp); epigingival 3D epithelium	E-cigarette aerosol	↑ levels of prostaglandin–E2 and ↑ cycloxygenase–2. ↑ oxidative/carbonyl and inflammatory responses, ↑ DNA damage, and ↓ histone deacetylase 2 (HDAC2) through RAGE–dependent mechanisms in gingival epithelium. Increased response in case of flavored e-cigarettes.	Sundar et al., 2016 (USA) [79]
▪ Primary human oropharyngeal mucosal cells	E-liquids with nicotine (2 fruit-flavored and 1 tobacco-flavored), and the corresponding base mixtures (free of nicotine and flavor)	Cytotoxic to oropharyngeal tissue. Significantly ↑ DNA fragmentation.	Welz et al., 2016 (Germany) [26]
▪ Normal epithelial cells: spontaneously transformed aneuploid immortal keratinocyte cell line from adult human skin (HaCaT); HNSCC cell lines: from a metastatic lymph node (UMSCC10B), and from a primary laryngeal tumor (HN30)	E-cigarette aerosol PG:VG (70%/30%) Flavors: “Classic Tobacco”, “Red American Tobacco” Nicotine: 12 mg/mL	Cytotoxic to epithelial cell lines. ↑ rates of apoptosis, ↑ rates of necrosis, independently of nicotine content. DNA strand break–induction.	Yu et al., 2016 (USA) [65]
▪ Primary human gingival fibroblasts	E-liquids (Two kinds: with and without nicotine)	Cytotoxic to cells. Both nicotine-containing and nicotine-free liquids induced ↑ reactive oxygen species (ROS) production.	Sancilio et al., 2015 (Italy) [75]
▪ Human periodontal ligament fibroblasts	Test solutions with components from E-liquids: lime-, hazelnut- and menthol-flavored liquids, nicotine, propylene glycol, and PBS as control group	Harmful effect of menthol additive on human periodontal ligament fibroblasts.	Willershausen et al., 2014 (Germany) [74]

Explanation: ↑—increase, ↓—decrease.

**Table 3:** The impact of e-cigarette liquid exposure on animal models (in vivo studies).

Animals	Characteristic of E-Liquid	Action	Reference
Female Wistar albino rats *n* = 16 (two groups) study group was exposed to vapor for 1 hour/day for 4 weeks	Electronic nicotine delivery system (ENDS)	Hyperplasia and metaplasia of the laryngeal mucosa of some rats but not significant statistically.	Salturk et al., 2015 (Turkey) [81]

**Table 4:** The impact of aerosol from e-cigarette on their users (in vivo studies)

Patients and Material	Characteristic of E-Liquid	Action	Reference
119 volunteers: 40 ± 1 in each of three cohorts—never smokers; tobacco smokers (smoking at least 10 cigarettes per day); e-cigarettes users Material: saliva	E-cigarette aerosol	Statistically significant ↑ abundance of *Veillonella* and *Porphyromonas* among e-cigarettes consumers. Highly ↑ interleukin (IL)-6 and ↑ IL-1β among e-cigarette users in comparison to non-users. E-cigarette users more susceptible to infection.	Pushalkar et al., 2020 (USA) [86]
90 volunteers: 45 former smokers and 45 E-cigarettes consumers. Material: oral mucosal lesions	E-cigarette aerosol	Oral mucosal lesions (a hairy tongue, nicotine stomatitis, and angular cheilitis) significantly more frequent among e-cigarettes users than in former smokers.	Bardellini et al., 2018 (Italy) [85]
59 volunteers: 20 e-cigarette users, 20 smokers, and 19 nonsmokers Material: saliva	E-cigarette aerosol	Endogenous NNN formation inside oral cavity. Mean concentration of NNN among e-cigarette consumers: 14.6 (± 23.1) pg/mL of saliva.	Bustamante et al., 2018 (USA) [83]
65 volunteers divided into three groups (non-smokers, tobacco smokers, e-cigarette users) Material: oral mucosa scrapings	E-cigarette aerosol	Prevalence of micronuclei significantly ↓ among e-cigarette users.	Franco et al., 2016 (Italy) [82]
10 volunteers immediately after vaping Material: buccal mucosa	E-cigarette aerosol (with and without nicotine)	↑ capillary perfusion of buccal mucosa (e-cigarette with nicotine).	Reuther et al., 2016 (UK) [84]

Explanation: ↑—increase, ↓—decrease.
